# An Organizing Framework for Translation in Public Health: The Knowledge to Action Framework

**Published:** 2011-02-15

**Authors:** Katherine M. Wilson, Teresa J. Brady, Catherine Lesesne, on behalf of the NCCDPHP Work Group on Translation

**Affiliations:** Epidemiology and Applied Research Branch, Division of Cancer Prevention and Control, National Center for Chronic Disease Prevention and Health Promotion, Centers for Disease Control and Prevention; Centers for Disease Control and Prevention, Atlanta, Georgia; Centers for Disease Control and Prevention, Atlanta, Georgia

## Abstract

A priority for the Centers for Disease Control and Prevention (CDC) is translating scientific knowledge into action to improve the public's health. No area has a more pressing need for translation than the prevention and control of chronic diseases. Staff from CDC's National Center for Chronic Disease Prevention and Health Promotion worked across disciplines and content areas to develop an organizing framework to describe and depict the high-level processes necessary to move from discovery into action through translation of evidence-based programs, practices, or policies. The Knowledge to Action (K2A) Framework identifies 3 phases (research, translation, and institutionalization) and the decision points, interactions, and supporting structures within the phases that are necessary to move knowledge to sustainable action. Evaluation undergirds the entire K2A process. Development of the K2A Framework highlighted the importance of planning for translation, attending to supporting structures, and evaluating the public health impact of our efforts.

## Introduction

As the nation's leading public health agency, the Centers for Disease Control and Prevention (CDC) not only conducts public health research but uses the findings to improve the public's health. Critical to CDC's success is enhancing the use of evidence-based practice by our constituents and partners. No area has a more pressing need for bridging research and practice than the prevention and control of chronic diseases. The World Health Organization (WHO) estimated in 2004 that chronic diseases accounted for 56% of deaths and 45% of the global burden of disease (1). In the United States, at least 80% of adults aged 65 years or older now have at least 1 chronic condition (eg, arthritis, diabetes, hypertension, heart disease) and obesity and its sequelae are threatening the health of future generations (2). Addressing the burden caused by these chronic health conditions is needed for the health of the nation's people and its economy, as health care spending is likely to increase with the aging of the population. One of the most efficient ways to use our limited public health dollars may be to apply "what we know works" (3).

To facilitate understanding of critical translation processes within CDC's National Center for Chronic Disease Prevention and Health Promotion (NCCDPHP), a group of scientists and practitioners from each of NCCDPHP's divisions and offices formed a workgroup, the Work Group on Translation (WGoT), to share translation-related experiences and observations (4). Because WGoT members came from various content areas, professional disciplines, and approaches to public health, it quickly became apparent that a common language and conceptualization were required to collaboratively expand our understanding of these processes. In this article, the term *translation* is used to mean the process and steps needed and taken to ensure effective and widespread use of evidence-based programs, practices, and policies. Thus *translation* is a term for putting knowledge from research or practice into action.

Several theories and frameworks exist to guide or explain aspects and processes involved in translation of evidence-based programs, practices, and policies. Syntheses of translation literature suggest complex processes are involved in diffusing evidenced-based innovations, including individual, organizational, and system-level characteristics that facilitate and hinder translation success (5-7). WGoT members developed an organizing framework informed by explicit frameworks (8-10), theoretical models (11), and tacit models in use by the various divisions and programs within NCCDPHP (12,13). We needed to create an organizing framework that conceptually would accommodate the various approaches to translation used across the center. Consequently, NCCDPHP's Knowledge to Action (K2A) Framework (Figure) and glossary (Box) were created to foster translation, communication, and collaboration across the center and within and across divisions. The purpose of this article is to present the resulting framework and discuss its use in planning and supporting translation in public health research and practice.

**Figure:**
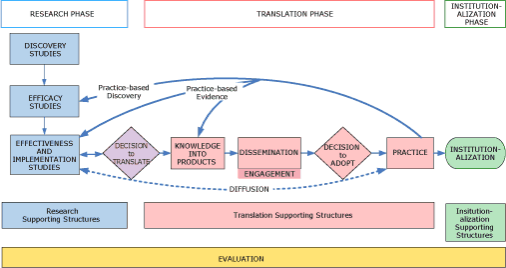
NCCDPHP Knowledge to Action Framework for Public Health.

**Box. Glossary for Knowledge to Action Framework**

**Research Phase**

*Discovery*: The original biomedical, behavioral, or epidemiologic factor that stimulated development of an intervention (1).

*Efficacy*: The extent to which the intended effect or benefits were achieved under optimal conditions (2).

*Effectiveness*: The extent to which the intended effect or benefits that were achieved under optimal conditions are also achieved in real-world settings, and the understanding of the processes by which research findings are put into practice (implementation research) (2).

*Research supporting structures*: Interrelated elements that enhance the capacity of an organization to effectively plan, implement, evaluate, and sustain the research phase of the intervention process, including marketing, training, technical assistance, financial resources, and organizational capacity (3).

**Translation Phase**

*Translation*: The process and steps needed or taken to ensure effective and widespread use of science-based programs, practices, and policies; a term for the entire process of putting research into practice. The term *translation* may also be used more narrowly to describe the process of making materials in an intervention linguistically appropriate.

*Decision to translate*: The decision to create an actionable product based on existing science-based knowledge or the decision to propel an evidence-based program, practice, or policy into widespread use.

*Knowledge into products*: A systematic process of turning scientific evidence and audience research into programs, policies, interventions, guidelines, tool kits, strategies, and messages that will assist and support audiences or users in putting science into practice.

*Dissemination*: A purposeful and facilitated process of distributing information and materials to organizations and individuals who can use them to improve health (2,4).

*Engagement*: The active participation and collaboration of stakeholders who can mobilize resources and influence systems to change policies, programs, and practices (5,6).

*Decision to adopt*: The decision at the organizational or community level to implement a program, policy, or practice (7,8).

*Practice*: Performing the tangible tasks and action steps to achieve public health objectives (9).

*Translation supporting structures*: Interrelated elements that enhance the capacity of each organization to effectively plan, implement, evaluate, or sustain the translation phase of the intervention process, including marketing, training, technical assistance, financial resources, and organizational capacity (3).

**Interactions Between Research and Translation Phases**

*Practice-based discovery*: Innovative field-based practices that lack data on their intended effects or benefits.

*Practice-based evidence*: Data from field-based practices that demonstrate achievement of intended effects or benefits.

*Diffusion*: The process through which an innovation spreads via communication channels over time among the members of a social system (4,7).

**Institutionalization Phase**

*Institutionalization*: The maintenance of an intervention (program, policy, or practice) as an established activity or norm within an organization, community, or other social system (10).

*Evaluation*: A systematic process for an organization to 1) improve and account for public health actions, and 2) obtain information on its activities, its impacts, and the effectiveness of its work to improve activities and describe accomplishments (11,12).

**References**

1BrownsonRCKreuterBAArringtonBATrueWR1211200697103Translating scientific discoveries into public health action: how can schools of public health move us forward?Public Health Rep1641670410.1177/003335490612100118PMC14977982FlayBRBiglanABoruchRFCastroFGGottfredsonDKellamS632005151175Standards of evidence: criteria for efficacy, effectiveness and disseminationPrev Sci1636595410.1007/s11121-005-5553-y3RobinsonKLDriedgerMSElliottSJEylesJ742006467476Understanding facilitators of barriers to health promotion practiceHealth Promot Pract1688550910.1177/15248399052789554LomasJ7031993226235226-35; discussion 235-7Diffusion, dissemination, and implementation; who should do what?Ann N Y Acad Sci819229910.1111/j.1749-6632.1993.tb26351.x5CDC/ATSDR Committee on Community EngagementAtlanta (GA)Centers for Disease Control and Prevention1997Coordinated school health programs and academic achievement: a systematic review of the literaturePrinciples of community engagement6FawcettSBPaine-AndrewsAFranciscoVTSchultzJARichterKPRichterKP2351995677697Using empowerment theory in collaborative partnerships for community health and developmentAm J Community Psychol885134510.1007/BF025069877RogersEM1983, 2003New York (NY)Free Press3rd edition and 5th editionDiffusion of innovations8OrlandiMA1611987119130Promoting health and preventing disease in health care settings: an analysis of barriersPrev Med382301010.1016/0091-7435(87)90011-99GreenLWKreuterMW2005New York (NY)McGraw-Hill4th editionHealth program planning: an educational and ecological approach10GlanzKRimerBKViswanathK2008San Francisco (CA)Jossey-Bass4th edition317Health behavior and health education: theory, research, and practice11Centers for Disease Control and Prevention48RR-1119992Framework for program evaluation in public healthMMWR Recomm Rep1049939712MattessichPW2003Saint Paul (MN)Amherst H. Wilder Foundation4th editionThe manager's guide to program evaluation: planning, contracting, and managing for useful results

The K2A Framework is not a causal or theoretical model but a schematic for processes that can be used by practitioners gathering practice-based discoveries or evidence (going from right to left in the framework diagram) and by researchers developing and testing interventions (going from left to right). The framework was designed to be applicable regardless of the disease, condition, or risk factor being addressed and regardless of the type of intervention being considered (ie, program, policy, or practice); to incorporate involvement of all actors in the research and practice communities (including scientists, administrators, policy makers, support systems, and practitioners); and to identify crucial points of interface between them (4). The K2A Framework reflects the developers' experience in the field, showing that public health practitioners and practitioner-generated innovations are needed for effective translation.

We recognize that each component in the translation process involves multiple decisions necessitating myriad smaller steps. We also recognize that although a framework on paper appears linear, translation processes are nonlinear and recursive (5,14). For the sake of parsimony, however, only major components and critical decision points and connections are included in the schematic.

## K2A Framework

### Three phases of the K2A Framework

The NCCDPHP K2A Framework identifies 3 phases in the overarching processes of moving from scientific discovery to routine public health practice: research, translation, and institutionalization. The research phase 1) includes developing and testing of scientific advances to determine their appropriateness for translation and 2) uses traditional definitions of efficacy, effectiveness, and implementation research. Biomedical or behavioral research and surveillance are needed to validate an approach or verify the efficacy of a program, practice, or policy. These occur before translation, but ideally occur with translation in mind (15). The translation phase incorporates the processes needed to ensure widespread implementation of evidence-based programs, practices, and policies. These processes include making the decision to translate, transforming scientific knowledge into actionable products, developing appropriate supporting structures, and disseminating evidence-based programs, practices, or policies to potential adopters. Implementation in practice depends on the communities, organizations, and practitioners making the decision to adopt and having sufficient supporting structures and resources to effectively move toward action. Effective translation of a program, practice, or policy is likely to follow a similar course, regardless of whether it originates in research or the field, although the specifics of the set of activities within each component will vary depending on the type of intervention. Ideally, successful translation processes lead to the institutionalization phase or maintenance of the program, practice, or policy as an established activity or norm within the community, organization, or social system (16). Processes throughout the translation framework combined with evidence that the efforts are cost-effectively achieving desired public health outcomes can support or inhibit the likelihood of institutionalization (17). In addition, environmental forces such as resource availability (fiscal and technical), quality supporting structures, stakeholder buy-in, and leadership support can affect the longevity of practice changes. Although the WGoT focused most of its attention on the translation phase, we recognize that institutionalization is the ultimate success of translation processes.

### Interactions between the research and translation phases

Inherent in successful translation is input from the practice community to the professionals involved in intervention research and development. This much-needed input is represented on the translation schematic by the practice-based discovery and practice-based evidence arrows. Field-based discovery highlights the opportunity to conduct efficacy and effectiveness studies on innovative field-based practices that lack data on their intended effects or benefits. Practice-based evidence returns field-based data to the professionals involved in effectiveness and implementation studies or those working to transform knowledge into products (18).

Few formal mechanisms exist for interactions among practitioners delivering interventions and the researchers developing and evaluating those interventions. A notable example of this interaction is the Active for Life initiative funded by the Robert Wood Johnson Foundation, which evaluated the effectiveness of 2 physical activity interventions. As part of that initiative, practitioners delivering the interventions provided feedback on translation issues to program developers. On the basis of the practitioner feedback, program developers reduced the reading level of program materials and produced a shorter version of 1 of the interventions, which facilitated its use in the field (19).

The decision to translate, the intentional decision to use translation processes to move a specific intervention into widespread use, is an essential transition point from the research phase to the translation phase. Integral to intentional translation efforts is a formal determination that the science base is adequate or that public health need suggests that it is time to act (eg, create some form of intervention). Evidence grading systems (20) provide metrics from trials and observational studies to determine when a specific intervention is ready for translation into the field. Although there are multiple decisions throughout the K2A Framework that advance translation, this explicit decision to translate is pivotal to moving purposefully from research to practice. This action is distinct from more passive attempts because it entails a thoughtful decision and active engagement of translation processes (11).

### Supporting structures

Supporting structures are interrelated elements undergirding all K2A processes. Each phase requires both general and intervention-specific structures such as organizational capacity, champions, staffing, financial resources, training, technical assistance, and intangibles such as leadership and political will (21). Structures to support interventions must be created and sustained to ensure quality practice change and institutionalization.

### Evaluation

Evaluation is fundamental to improving translation processes; it provides information on whether evidence-based interventions are reaching the people who need them and it assesses our success in achieving desired health outcomes (22,23). Evaluation as used in the K2A Framework is multifaceted, present throughout the entire translation process, and inherent in each component. The framework does not attempt to dictate how evaluation of translation efforts should be designed or conducted. Although fundamental evaluation questions may evolve for processes across translation, evaluation strategies and methods need to be unique to context and situation. The evaluation bar in the K2A Framework reminds users to incorporate appropriate evaluation activities and measures throughout translation processes.

## Discussion

### Planning for translation

The entire public health system, from researcher to practitioner, needs to be involved and accountable for putting scientific knowledge into action. Researchers should keep feasibility of implementation clearly in mind when developing interventions so as to avoid developing interventions that are too resource-intensive or require organizational commitment that is unrealistic. For example, a physical activity intervention that requires delivery by physical therapists might not be feasible in senior centers with limited resources. Practitioners should carefully review the literature and other resources such as online clearinghouses when selecting evidence-based interventions to use. If using a home-grown intervention in the field, practitioners should develop evaluation plans, monitor program outcomes, and assist in building the evidence base. Gathering data on these field-based interventions exemplifies the multiple responsibilities for translation and the bidirectional nature of the framework.

One use of the framework in planning is as a reminder of the comprehensive process of translating knowledge into action. We can research or evaluate a specific component of intervention development or translation without treating it as an independent process, because we have a sense of where these findings fit in the overall schema of K2A (24).

### Attending to supporting structures

In all 3 phases of the K2A Framework, structures and systems must be put into place to support use of evidence-based interventions and practices in the field. Research-related supporting structures such as research grants and training fellowships are vital to the creation of scientific knowledge. Applying that knowledge during the translation and institutionalization phases requires both general and intervention-specific supports. One supporting structure might be the requirement by funding agencies for applicants to use an evidence-base intervention, either a particular intervention or 1 chosen from a menu of acceptable evidence-based interventions. Another supporting structure could include readily available training, technical assistance, and well-researched marketing materials. Organizational capacity, political will, and financial resources are all essential to the success of any intervention, as are intervention-specific dissemination packages, training, and technical assistance (14). Dissemination packages such as how-to guides are of limited use, however, in the absence of organizational capacity. Similarly, technical assistance will produce limited benefits if financial resources or political will to carry out the intervention are absent. Support from state, local, tribal, and federal public health officials is vital to establishing organizational and workforce norms that reinforce motivation to use evidence.

## Evaluating Public Health Impact

Although using evidence-based interventions will result in changes in population-based health status, these changes may not be immediate (25). In our view, translation efforts in public health are achieving their purpose if 1) effective programs, practices, and policies are implemented on a wide scale and with quality and fidelity and 2) these efforts are sustained over time (ie, institutionalized) (6,26,27).

Collectively, we must ensure that our investments are resulting in widespread use of evidence-based programs, practices, and policies by our public health partners. The RE-AIM framework (9), for example, gives us some metrics on the extent of translation and public health impact. The NCCDPHP K2A Framework, which emphasizes translation processes, can help identify interim milestones or indicators of progress in translation. For example, the number of corporations that adopt a policy or the number of people who are trained to deliver an intervention can serve as interim milestones on the path of successful translation. Because translation involves a complex set of processes, indicators of success and quality should routinely be used in funding opportunity announcements, reporting criteria, and funding policies. The K2A Framework guides users to consciously consider the translation processes from which specific indicators relevant to the innovation being translated can be identified.

## Conclusion

The NCCDPHP K2A Framework and Glossary were created to meet the need for a common language and conceptual framework to allow public health researchers and practitioners to work productively together. Collaboration among researchers, practitioners, and other professionals is essential to successfully move scientific knowledge to widespread public health practice and to increase the influence of practice-based knowledge on research. The K2A Framework will be a useful organizing structure to plan for translation, to invest in supporting structures, and to hold ourselves accountable for successful translation of evidence-based programs, practices, and policies for improving the public's health.
